# The remaining parameters of patellar instability could be affected for osteoarthritic change after medial patellofemoral ligament reconstruction with or without anteromedialization of the tibial tubercle osteotomy for patellar instability: a retrospective cohort study

**DOI:** 10.1186/s12891-022-06100-3

**Published:** 2023-01-23

**Authors:** Yusuke Hashimoto, Kazuya Nishino, Tomihara Tomohiro, Shuko Tsumoto, Hiroaki Nakamura

**Affiliations:** 1 Department of Orthopaedic Surgery, Osaka Metropolitan University Graduate School of Medicine, Osaka, Japan; 2 Department of Orthopaedic Surgery, Shimada Hospital, Habikino, Japan

**Keywords:** Medial patellofemoral ligament reconstruction, Postoperative osteoarthritis, Anteromedialization, Tibial tubercle osteotomy

## Abstract

**Background:**

In literature, studies evaluating the factors associated the postoperative progression of patellofemoral (PF) osteoarthritis (OA) following patellar stabilization surgery are limited. This study aimed to compare the clinical outcomes after medial patellofemoral ligament reconstruction (MPFLR) as an isolated procedure (iMPFLR) and in combination with anteromedialization (AMZ) of the tibial tubercle osteotomy (TTO) and investigate the factors related to the postoperative progression of PFOA after patellar stabilization surgery.

**Methods:**

Between 2009 and 2020, 30 knees of 23 consecutive patients underwent MPFLR with or without AMZ, using an autologous semitendinosus tendon graft; they were followed up for more than 2 years in the retrospective nature of the study. iMPFLR was performed in cases of recurrent patellar dislocation with normal tibial tubercle-trochlear groove (TT-TG) distance and no PFOA, and MPFLR+AMZ was performed for cases of excessive TT-TG distance, preoperative PFOA of recurrent patellar dislocation, or habitual patellar dislocation. Clinical findings and radiographs of the PF joint were evaluated pre- and postoperatively with PF alignment parameters and PFOA and were compared between surgical procedures. Factors for the postoperative progression of PFOA were compared between the OA progression and non-progression groups.

**Results:**

Postoperative clinical score, radiographic parameters except for sulcus angle, TT-TG distance, and progression of PFOA were not significantly different between the iMPFLR and MPFLR+AMZ groups. Postoperative lateral patellar displacement (*p* = 0.001) and congruence angle (*p* = 0.017) were significantly different between the OA progression and non-progression groups.

**Conclusion:**

Similar to MPFLR for recurrent cases, MPFLR with AMZ can improve the clinical and radiographic outcomes in severe cases. The remaining parameters of patellar instability could be affected in the postoperative progression of PFOA after MPFL reconstruction with or without AMZ of TTO for patellar instability.

## Background

Patellar instability is a common knee pathology during growth that affects daily activities and participation in sports. Patellofemoral (PF) stability depends on the relationship between osseous and soft tissue anatomy, in addition to dynamic muscular control and overall limb alignment. Although the etiology of patellar instability is multifactorial, the goal of surgery is to stabilize the patella, restore normal kinematics, and optimize load transmission through the joint in cases of failed conservative treatments. The medial patellofemoral ligament (MPFL) is a major passive restraint against lateral patellar forces that prevents lateral patellar dislocation during early flexion. MPFL reconstruction (MPFLR) has been shown to be effective in restoring stability for recurrent patellar dislocations in numerous studies [1–3]; however, isolated MPFLR (iMPFLR) is prone to failure in patients with excessive lateralized tibial tubercle, severe trochlea dysplasia, and patella alta [4–6]. Habitual patellar dislocation is rare, and there is still much controversy regarding its etiological factors and treatment, especially regarding the choice of the surgical method [7, 8]. Osteoarthritic (OA) changes in the PF joint are often observed after patellar dislocation. Patients with OA changes in the PF joint and patellar instability often experience both instability and pain in their knees. However, there is no consensus treatment for patellar instability with OA changes in the PF joint [9]. A combined approach is indicated in patients with patellar instability, particularly in severe cases of patellar instability. Anteromedialization (AMZ) of the tibial tubercle osteotomy (TTO) is an effective distal procedure for stabilizing the patella by decreasing the angle of patellar engagement and the pressure of the patellofemoral joint [10, 11]. However, information on combined treatment is sparse, especially in the setting of multiple factors, such as excessive tibial tubercle (TT)-trochlear groove (TG) distance and preoperative OA change in the PF joint of recurrent patellar dislocation or habitual patellar dislocation. Surgical treatment for patellar instability sometimes leads to an increased incidence of OA in the patellofemoral joint postoperatively at long-term follow-up [12–14]. However, evidence for the factors associated with the postoperative progression of PFOA is limited. Therefore, this study aimed to investigate the clinical outcomes after iMPFLR and simultaneous MPFLR and AMZ of the TTO (MPFLR+AMZ) in cases of excessive TT-TG distance and preoperative OA change in the PF joint of recurrent patellar dislocation or habitual patellar dislocation. Additionally, factors related to PFOA progression were analyzed radiographically.

the hypothesis is that (1) the clinical result would not differ between iMPFLR and MPFLR+AMZ, and (2) the remaining parameters of patellar instability after surgery could be factors for the progression of PFOA after patellar stabilization surgery.

## Material and methods

We retrospectively reviewed the medical records from August 2009 to January 2020, including the radiographic records of patients treated with MPFL reconstruction with or without AMZ of the TTO by a single surgeon. The inclusion criterion was surgical cases of recurrent or habitual patellar dislocation including cases of *trochlear dysplasia and patella alta.* The exclusion criteria were as follows: (1) congenital patella dislocation, (2) less than 2 years of follow-up, (3) lack of medical records, (4) other surgical procedures, and (5) previous knee surgery. The patients were divided into two surgical groups (iMPFLR and MPFLR+AMZ) according to the therapeutic protocol. The indication for iMPFLR was patellar instability as recurrent dislocation with a TT-TG distance of less than 20 mm and no joint space narrowing of the PF joint or open physis. The indications for MPFLR+AMZ were patellar instability as greater than 20 mm of TT-TG distance, joint space narrowing of the PF joint, or habitual patellar dislocation. The TT-TG distance was identified using preoperative CT scans. Narrowing of the PF joint space was identified as more than stage 1 in Iwano’s classification [15]. Based on previous studies, recurrent patellar dislocation was defined as two or more episodes of lateral patellar dislocation [16]. Habitual patellar dislocation was identified as a lateral dislocation of the patella each time the knee was flexed, returning to the midline with the extension of the knee [7].

### Surgical procedure

#### Isolated MPFLR

**Fig. 1:**
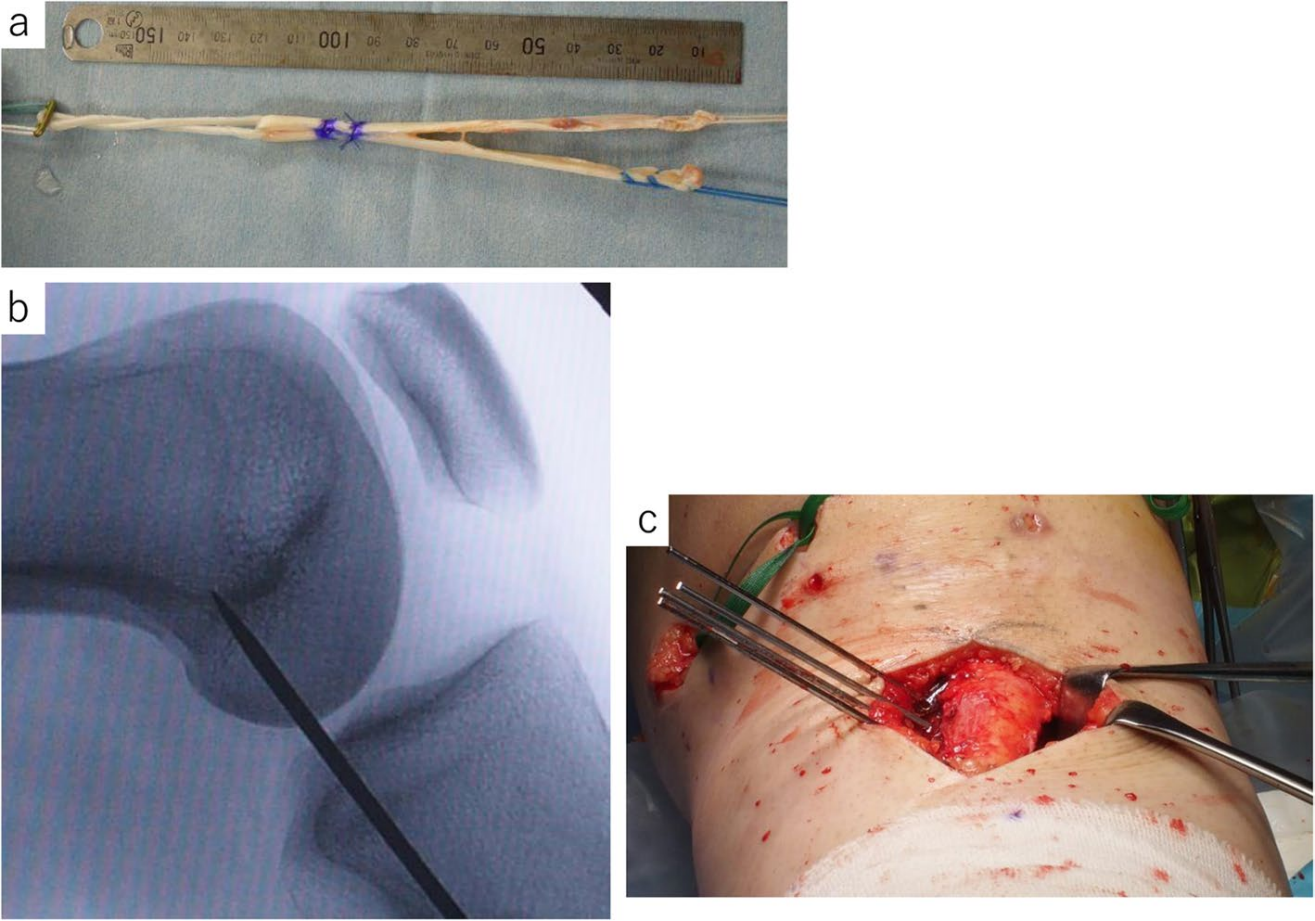
**a** A free autologous semitendinosus graft with medial patellofemoral ligament reconstruction. The semitendinosus tendon was double-looped over the Endobutton CL and graft ends were sutured with No. 3 sutures in Krackow stitch fashion. **b** Fluoroscopic finding of the tunnel position. The femoral tunnel was located using the medial epicondyle and adductor tubercle as anatomical landmarks and fluoroscopy was used as confirmation for appropriate positioning on the lateral image by Schöttle. **c** Anteromedialization of the tibial tubercle osteotomy. A longitudinal incision was made starting from the tibial tubercle to the distal 7 cm. After marking the medial side of the tibia, Kirschner wires were used from medial to lateral, angling posteriorly as the osteotomy guide

The patient was positioned supine on a radiolucent table and diagnostic arthroscopic surgery was performed. Arthroscopic lateral release was performed with a thermal device for all cases. After arthroscopic surgery, MPFLR was performed with a free autologous semitendinosus graft. The semitendinosus tendon was double-looped over the Endobutton CL (Smith and Nephew Inc. Mansfield, MA), and graft ends were sutured with No. 3 Elp sutures (Akiyama Inc., Japan) in a Krackow stitch fashion (Fig. 1a). After harvesting the semitendinosus tendon, a 3-cm skin incision was made over the medial margin of the patella and medial epicondyle of the femur. The femoral tunnel was located using the medial epicondyle and adductor tubercle as anatomical landmarks, and fluoroscopy was used to confirm appropriate positioning on the lateral image [17] (Fig. 1b). A K-wire was placed in the femur to hold the graft in the antero-proximal direction to avoid a posterior cortical blowout. The guidewire was over-drilled to a depth of 30 mm using a drill bit that matched the diameter of the graft in 0.5 mm steps. The graft was inserted into the femoral bone tunnel and the EndoButton was flipped on the femoral cortex. The graft was then brought between the deep fascia and capsule of the knee joint and out through the incision over the medial margin of the patella. For patellar site fixation, a GII anchor (Depuy-Mitek, Raynham, MA) was inserted into patella between the two tailed graft and tied with both grafts. After fixation with anchor, the graft was sewn with nonabsorbable threads to the patellar periosteum and medial parapatellar retinaculum at the center of the patellar articular surface. The knee joint was flexed at 60° to ensure patellar engagement in the trochlea to prevent medializing the patella and fix the graft at its longest length [18].

#### MPFLR with AMZ of the TTO

After arthroscopic surgery of the lateral release procedure, AMZ of the TTO was performed according to the techniques described previously [19, 20]. A longitudinal incision was made starting just medial to the TT. The osteotomy site was marked on the medial aspect of the tibia, starting proximally next to the tubercle at a depth of 1.0–1.5 cm and continuing distally 7 cm. As the mark was extended distally, the bony shingle was thinned to a thickness of 2–4 mm at the most distal aspect to avoid creating a step cut. This allowed the shingle to hinge and rotate medially, with minimal prominence. After marking the medial side of the tibia, Kirschner wires were used from medial to lateral, angled posteriorly as the osteotomy guide (Fig. 1c). A bone cut was made using a chisel. The tibial tuberosity was anteromedially transferred to achieve medialization, estimated from the preoperative CT images. The transferred tuberosity was fixed with two 4.5-mm fully threaded cortical screws (Meira, Nagoya,Japan) using a lag technique and countersinking to prevent screw-head prominence. Distance between the original position and transferred position of the lateral cortex of the tibial tuberosity was measured and the average and standard deviation (SD) were 9.4 ± 1.9 (range, 6–13) mm. After fixing the TT, the semitendinosus tendon was harvested, and MPFLR was performed as above.

### Rehabilitation protocol

The same rehabilitation program was performed in both groups. Patients were immobilized with a brace for 1 week and then limited to a knee range of motion of 0–120° for 3 weeks, followed by partial weight-bearing for 5 weeks without a hinged knee brace locked in full extension. These patients were permitted to jog at 3 months post-surgery and return to sports activities 6 months post-surgery.

### Clinical evaluation

The clinical assessment consisted of evaluating the patients for apprehension test and Kujala scores preoperatively and at the final follow-up [21].

### Radiographic evaluation

Plain radiography of the knee, including anteroposterior, lateral, and Merchant’s views, was performed preoperatively and at the final follow-up. Merchant’s view radiographs were used to measure the sulcus, congruence, and tilting angles, and lateral patellar displacement to radiographically evaluate the presence of patellar tracking defects [22]. The sulcus angle was defined as the angle between the lines passing by the deepest trochlear point and the anterior knee condyles. The congruence angle was defined as the angle between the two lines: one bisecting the sulcus angle and one connecting the deepest point of the trochlear groove and the lowest point of the patellar ridge. The tilting angle was defined as the angle between the two lines; one was the transverse axis of the patella and the other was the line tangent to the top of the medial and lateral femoral condyles. Lateral patella displacement was defined as the distance between the line perpendicular to the trochlear surface passing by the most medial patellar portion and the perpendicular line passing by the apex of the medial anterior condyle.

Trochlear dysplasia was measured using lateral radiographs according to the Dejour classification. In the lateral view, the Insall–Salvati ratio (ISR) was used to assess patellar height. The TT-TG distance was assessed using preoperative CT images to detect lateral malposition of the tibial tuberosity. PFOA severity was evaluated preoperatively and at the final follow-up using Iwano’s classification on the skyline view [15].

**Fig. 2:**
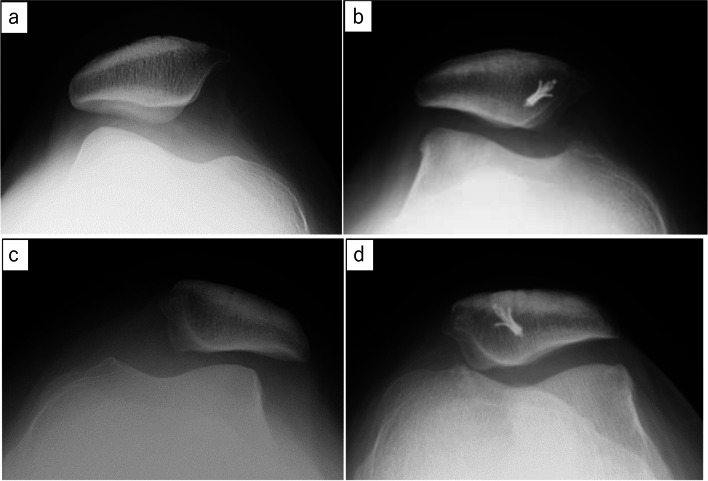
Representative pre and postoperative X-ray in the OA progression and non-progression case who underwent isolated medial patellofemoral ligament reconstruction (iMPFLR) and MPFLR with anteromedialization of the tibial tubercle osteotomy (MPFLR+AMZ). **a** Stage 0 of preoperative X-ray of a left knee at 16 years of age before isolated medial patellofemoral ligament reconstruction; **b** Stage 1 of postoperative X-ray 5 years following iMPFLR. **c** Stage 1 of preoperative X-ray of a right knee of a 35-year-old woman before MPFLR+AMZ. **d** Stage 1 of postoperative X-ray 7 years following MPFLR+AMZ

OA progression group was defined by worse PFOA in the final follow-up than in the preoperative stage (Fig. 2a, b). The non-progression group was defined by the same or improved staging (Fig. 2c, d).

### Statistical analysis

Preoperative and postoperative parameters (age, sex, BMI, pre/postoperative Kujala score, sulcus angle, congruence angle, tilting angle, lateral patellar displacement, trochlea dysplasia, Dejour classification, ISR, TT-TG distance, and Iwano’s classification) were compared between the iMPFLR and MPFLR+AMZ groups or between the reccurent and habitual groups. The Wilcoxon signed-rank test and paired *t-test* were used to compare the pre- and post-operative data of each group. The Student’s t-test was used to compare continuous variables (age, Kujala score, sulcus angle, congruence angle, tilting angle, lateral patellar displacement, ISR, and TT-TG distance) between the iMPFLR and MPFLR+AMZ groups, and the OA progression and non-progression groups. The χ^2^ test or Fisher’s exact test was used to evaluate categorical variables (sex, trochlea dysplasia, Dejour classification, surgical procedure, and Iwano classification). The significance level was set at *P* < 0.05. Intraclass correlation coefficients were assessed by two orthopedic surgeons (KN and YH) to measure the intra- and inter-observer reliabilities for X-ray measurements of the lateral shift displacement and congruence angle. Measurements were performed at two separate points 1 month apart. The strength of agreement was interpreted as follows: 0.80, almost perfect agreement; 0.61–0.80, substantial agreement; 0.41–0.60, moderate agreement; 0.21–0.40, fair agreement; and ≤ 20, slight agreement. The intra- and inter-observer reliabilities of lateral patella displacement were 0.899 and 0.862, respectively, and those of congruence angle were 0.925 and 0.943. A power analysis was performed with the power (1-beta), a, difference and SD set at 0.8, 0.05, 5.53, and 2.96, respectively, according to lateral patella displacement. Analysis revealed that a minimum of 20 patients was required for the Wilcoxon test to detect a difference between the group with and without progression of PF osteoarthritis.

. EZR software version 1.38 (Saitama Medical Center, Jichi Medical University, Saitama, Japan) was used for all the analyses.

## Results

### Demographic data

**Fig. 3:**
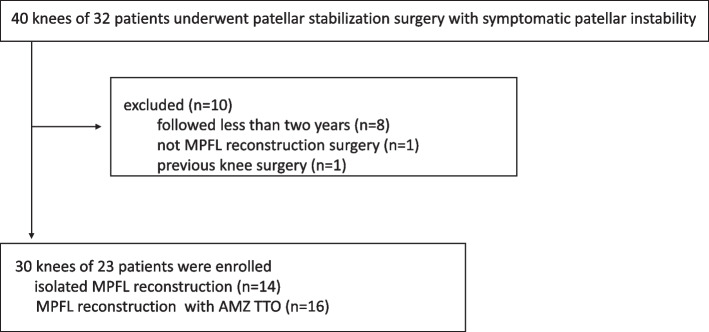
Flowchart of patient inclusion in this retrospective analysis

Thirty-two patients (40 knees) who underwent surgery for patellar instability were retrospectively recruited to participate in this study. Among these, eight knees of seven patients who were followed up for less than 2 years, 1 patient with other surgical procedures, and 1 patient with previous knee surgery were excluded. The remaining 30 knees of the 23 patients were included in this study (Fig. 3).

The mean ± SD age of the patients was 24.0 ± 10.0 years and the mean follow-up period was 5.0 ± 2.9 (2–12) years. iMPFLR was performed in 14 knees; 13 knees showed TT-TG distances of less than 20 mm and one knee showed with open physis. MPFLR in combination with AMZ of the TTO was performed in 16 knees; 4 knees showedcases of TT-TG distances of more than 20 mm, 6 knees showedt with PF joint space narrowing (2 of stage1, 2 of stage2, 1 of stage3, 1 of stage4), and 6 knees showed habitual instability. Preoperative demographic data of age, OA grade, trochlea dysplasia, congruence angle, sulcus angle, tilting angle, lateral patellar displacement, and TT-TG distance were significantly different between the iMPFLR and MPFLR+AMZ groups (Table 1). The aforementioned observations could have been the result of surgical indications.

**Table 1:** Comparison of the preoperative features between iMPFL and MPFL+AMZ groups

	iMPFL *N* = 14 (SD or %)	MPFL+AMZ *N* = 16 (SD or %)	*P* value
Age	19.6 (6.3)	27.8 (11.2)	.023
Gender, male/ female	4 (28.6) / 10 (71.4)	4 (25.0)/ 12 (75.0)	1.00
Side (L/R)	8 (57.1) / 6 (42.9)	8 (50) / 8 (50)	.730
Iwano OA grade (0/1/2/3/4), n	14/0/0/0/0	3/9/2/1/1	<.001
Trochlea dysplasia	8 (57.1)	16 (100)	.005
Dejour classification (A/B/C/D)	5/2/0/1	3/6/0/7	.162
Congruence angle, degree	32.3 (8.0)	58.8 (23.1)	<.001
Sulcus angle, degree	140.1 (3.4)	154.3 (15.8)	.003
Tilting angle, degree	31.4 (7.2)	50.7 (31.1)	.031
ISR	1.19 (0.17)	1.19 (0.30)	.938
Lateral patella displacement, mm	15.8 (4.8)	24.6 (12.5)	.020
TT-TG distance, mm	17.9 (3.0)	21.0 (3.1)	.010

*SD* standard deviation, *OA*osteoarthritis, *ISR* Insall–Salvati ratio, *TTTG* tibial tubercle-trochlear groove

### Clinical outcomes

No re-dislocation case and three cases with positive apprehension test (2 of iMPFLR, 1 of MPFLR+AMZ) results developed. Progression of PFOA was observed in four cases (3 of iMPFLR, 1 of MPFLR+AMZ. The Kujala score was improved significantly from preoperatively to postoperatively in both groups (Table 2). There was no statistically significant difference in the Kujara score between the two groups postoperatively (*P* = 0.652) (Table 3).

**Table 2:** Comparison between pre- and post-operative features

	iMPFL			MPFL+AMZ		
	Preoperative (SD)	Postoperative (SD)	*P* value	Preoperative (SD)	Postoperative (SD)	*P* value
Kujala score	68.4 (10.1)	94.9 (5.9)	<.001	55.5 (9.3)	93.8 (6.6)	<.001
Congruence angle, degree	32.3 (8.0)	2.9 (11.6)	<.001	58.8 (23.1)	−4.8 (34.0)	<.001
Sulcus angle, degree	140.1 (3.4)	139.6 (3.3)	.511	154.3 (15.8)	153.6 (15.7)	.704
Tilting angle, degree	31.4 (7.2)	18.8 (4.6)	<.001	50.7 (31.1)	15.1 (9.4)	<.001
ISR	1.19 (0.17)	1.13 (0.15)	.037	1.19 (0.30)	1.02 (0.23)	.001
Lateral patella displacement, mm	15.8 (4.8)	2.5 (2.8)	< .001	24.6 (12.5)	1.3 (3.9)	<.001
TTTG	17.9 (3.0)	16.2 (2.4)	.003	21.0 (3.1)	13.7 (3.3)	<.001
Iwano OA grade (0/1/2/3/4), n	14/0/0/0/0	11/2/1/0/0	1.00	3/9/2/1/1	3/10/3/0/0	<.001

*SD* standard deviation, *ISR* Insall–Salvati ratio, *TTTG* tibial tubercle-trochlear groove, *OA* osteoarthritis

**Table 3:** Comparison of the postoperative features between iMPFL and MPFL+AMZ groups

	Total *N* = 30 (SD or %)	iMPFL *N* = 14 (SD or %)	MPFL+AMZ *N* = 16 (SD or %)	*P* value
Kujala score	94.3 (6.2)	94.9 (6.0)	93.8 (6.6)	.652
Congruence angle, degree	− 1.2 (26.0)	2.9 (11.7)	−4.8 (34.0)	.425
Sulcus angle, degree	147.1 (13.5)	139.6 (3.3)	153.6 (15.7)	.003
Tilting angle, degree	16.8 (7.6)	18.8 (4.6)	15.1 (9.4)	.196
ISR	1.07 (0.20)	1.13 (0.15)	1.02 (0.23)	.128
Lateral patella displacement, mm	1.8 (3.4)	2.5 (2.8)	1.3 (3.9)	.341
TTTG	14.8 (3.1)	16.2 (2.4)	13.7 (3.3)	.036
Iwano OA grade (0/1/2/3/4), n	14/12/4/0/0	11/2/1/0/0	3/10/3/0/0	.004

*SD* standard deviation, *ISR* Insall–Salvati ratio, *TTTG* tibial tubercle-trochlear groove, *OA* osteoarthritis

### Radiographic outcomes

The congruence angles, tilting angle and lateral patella displacement of the participants in the iMPFLR and MPFLR+AMZ groups were significantly improved from preoperatively to postoperatively. (Table 2) Postoperative data of congruence angle, tilting angle, and lateral displacement were not significantly different between the iMPFLR and MPFLR+AMZ groups (Table 3), while the postoperative congruence angle, ISR and tilting angle were significantly different between the recurrent and habitual groups (Table 4).

**Table 4:** Comparison of the postoperative features between recurrent and habitual groups

	Recurrent *N* = 24 (SD or %)	Habitual *N* = 6 (SD or %)	*P* value
Kujala score	93.2 (6.6)	98.2 (2.0)	.086
Congruence angle, degree	5.6 (17.9)	−28.3 (36.5)	.003
Sulcus angle, degree	141.9.6 (8.1)	168.0 (15.7)	<.001
Tilting angle, degree	15.0 (6.6)	24.2 (7.7)	.006
ISR	1.14 (0.15)	0.83 (0.17)	<.001
Lateral patella displacement, mm	2.3 (2.8)	0.1 (5.2)	.175
TTTG	15.3 (3.0)	12.9 (3.2)	0.103
Iwano OA grade (0/1/2/3/4), n	13/7/4/0/0	1/5/0/0/0	.077

*SD* standard deviation, *ISR* Insall–Salvati ratio, *TTTG* tibial tubercle-trochlear groove, *OA* osteoarthritis

### Factors related to the progression of PFOA

Regarding the progression of PFOA following patellar stabilization surgery, postoperative lateral patellar displacement (*P* = 0.001) and congruence angle (*P* = 0.017) were significantly different between the OA progression and non-progression groups (Table 5).

**Table 5:** Comparison of the pre- and postoperative features between OA progression and non-progression groups

	OA progression (−) *N* = 26 (SD or %)	OA progression (+) *N* = 4 (SD or %)	*P* value
Age	25.0 (10.2)	17.5 (6.4)	.167
Gender, male/ female	6 (23.1)/ 20 (76.9)	2 (50)/ 2 (50)	.284
BMI	23.4 (3.7)	24.8 (2.5)	.478
Follow-up, year	4.8 (2.9)	6.2 (3.4)	.379
Reccurent / habitual	20 (76.9)/ 6 (23.1)	4 (100)/ 0 (0)	.557
Preoperative			
Kujala score	61.5 (11.6)	61.2 (13.0)	.964
Congruence angle, degree	47.0 (23.5)	43.0 (9.6)	.745
Sulcus angle, degree	149.1 (14.1)	138.5 (4.2)	.150
Tilting angle, degree	44.5 (25.6)	23.5 (37.0)	.117
ISR	1.18 (0.25)	1.27 (0.23)	.485
Lateral patella displacement, mm	21.2 (11.0)	15.9 (5.0)	.352
TTTG	19.4 (3.5)	20.9 (2.7)	.436
Iwano OA grade (0/1/2/3/4), n	14/8/2/1/1	3/1/0/0/0	1.00
Postoperative			
Kujala score	93.8 (6.6)	97.5 (2.1)	.274
Congruence angle, degree	−5.5 (24.0)	27.0 (22.0)	.017
Sulcus angle, degree	148.3 (14.1)	139.5 (3.9)	.233
Tilting angle, degree	16.8 (7.8)	17.0 (7.4)	.964
ISR	1.06 (0.20)	1.19 (0.16)	.233
Lateral patella displacement, mm	1.1 (3.0)	6.7 (2.2)	.001
TTTG	14.5 (3.1)	16.6 (2.9)	.209
Iwano OA grade (0/1/2/3/4), n	14/10/2/0/0	0/2/2/0/0	.018

*SD* standard deviation, *ISR* Insall–Salvati ratio, *TTTG* tibial tubercle-trochlear groove, *OA* osteoarthritis

## Discussion

The most important findings of this study are that the clinical and radiographic results showed no difference between iMPFLR and MPFLR+AMZ in severe cases; both methods achieved good clinical outcomes. However, persistent lateral patellar displacement was a risk factor for postoperative progression of PFOA over a minimum follow-up period of 2 years following patellar stabilization surgery.

iMPFLR is a safe and efficient surgical procedure, with a low failure rate, as shown in a long-term study [1, 2]. Otherwise, trochlear dysplasia and excessive lateralized tibial tuberosity are well-known risk factors for recurrence after first-time patellar dislocation and MPFLR failure [4, 5, 23–25]. Overtension of graft for MPFLR, including malposition of femoral tunnel placement, can induce an increase in pressure of the PF joint and lead to failure [26–28]. Severe cartilage injuries of the PF joint were identified as reasons for revision surgery after iMPFLR [29]. Potential anatomical abnormalities are one of the major reasons for adolescents to develop habitual patella dislocation, such as femoral trochlear dysplasia, TT-TG distance increase, lateral soft tissue contracture, and medial soft tissue relaxation [30]. AMZ of the TTO is a common treatment option for several patellofemoral joint disorders including chondromalacia, patellofemoral arthritis, and patellar instability. It is effective in severe cases of patella instability, such as excessive lateralized tibial tuberosity, trochlear dysplasia, preoperative OA change, and habitual patellar dislocation [20, 31–33]. AMZ of the TTO can modify tracking and/or patellofemoral contact forces to affect the unloading of chondral defects of the patella or trochlea, correct multiplanar suboptimal alignment, or be used in conjunction with soft-tissue stabilization procedures for instability [34–36].

In this study, the clinical outcome of MPFLR, with and without AMZ of the TTO, showed no significant difference at a minimum of 2 years of follow-up. This result is similar to that of a previous study evaluating mid-term [37, 38] and long-term clinical outcomes [39]. Mulliez et al. [37] investigated the clinical outcomes after iMPFLR and in association with AMZ of the TTO (in case of patella alta or an excessive TT-TG) for patellar instability and concluded that MPFLR with or without transposition of the tibial tubercle is safe and effective with clinically no significant difference after a 2-year follow-up, and a concomitant tuberosity transposition is useful in selected patients. Neri et al. [39] reported that MPFLR, whether isolated or associated with a TT medial or distal transfer for patients with excessive TT-TG distance, provides good long-term clinical and radiological outcomes with a low rate of recurrence. Tscholl et al. [38] compared the effectiveness of iMPFLR and MPFLR with TT medial or distal transfer for patella alta or excessive lateralized tibial tuberosity, in treating recurrent patellar dislocation and MPFLR, with and without TTO, was reported a reliable treatment option for recurrent patellar dislocation at the mid-term follow-up. Song et al. [40] demonstrated that the outcomes of MPFLR, with or without TTO, to treat recurrent or habitual patellar dislocation with an excessive TT-TG distance ranging from 16 to 20 mm appeared similar. In the present study, the indication for osteotomy was not only excessive TT-TG distance (> 20 mm) but also preoperative OA change and habitual patellar dislocation. Our indication was more severe than that in previous studies. The postoperative clinical results were the same, indicating that AMZ of the TTO was suspected to be effective in severe cases, such as excessive lateralized tibial tuberosity, preoperative OA change, and habitual dislocation.

Although it has been shown that patellar stabilization surgeries are associated with a lower risk of recurrent dislocations, they may bear a risk of subsequent PF joint degeneration. Therefore, these surgical techniques are considered to be risk factors for late OA due to increased patellofemoral contact pressure and changes in knee joint loading [41, 42]. Increased prevalence and progressive PF degeneration have been reported in patients with excessive lateralized tibial tuberosity, patellar tilt, and trochlear dysplasia [9, 43]. Nakagawa et al. [13] reported that definite osteoarthritic changes were detected in radiographs of 13 of 31 knees (42%) after the Elmslie–Trillat procedure, with a mean follow-up of 161 months. Nomura et al. [44] reported that only two of 24 knees (8.3%) had definite OA changes in radiographs after MPFLR, with a mean follow-up of 11.9 years. Shimizu et al. [45] reported that three of 20 knees (15%) had OA changes postoperatively following MPFLR. In the present study, four of 30 knees (13.3%) had postoperative OA changes. There was no difference between the surgical procedures; however, postoperative lateral patellar displacement (*P* = 0.016) and congruence angle (*P* < 0.001) were significantly different between the OA progression and non-progression groups. In this study, the patellar position remained lateral, and a lateral patellar position may lead to increased contact pressure to the lateral facet of the trochlea as previously described [9]. Giesler et al. [46] reported that trochlear dysplasia parameters, TT-TG distance, and postoperative persistent high lateral patellar tilt were risk factors for progressive knee joint degeneration after MPFLR. This suggests that the remaining parameters of patellar instability could be affected for the progression of PFOA after patellar stabilization surgery. Adequate patellar position was recommended at least during surgery to avoid progression of PFOA in long term follow-up period.

### Limitations

This study had several limitations. First, this retrospective cohort study included a small number of patients. Therefore, unseen variables could have introduced bias into the results. For example, we performed power analysis for lateral patellar displacement because of the significant difference between the OA progression and the non-progression group. There were no other observed differences between the two groups, which may indicate the lack of power in these parameters. Moreover, the progression of PFOA may have been underestimated because of the short follow-up period. Second, the inclusion criterion was broad with respect to the severity of cases. This study included patients with mild recurrent patellar instability as well as severe cases, such as excessive TT-TG distance, preoperative OA change in the PF joint of recurrent patellar dislocation, and habitual patellar dislocation. Third, no second-look arthroscopy was performed, and not all patients underwent MRI examination to assess cartilage status. Therefore, the present findings provide the “best-case scenario” for the progression of OA, and the actual progression of cartilage degeneration may be higher. Fourth, the parameters of patellar instability were evaluated with radiographic examination, not CT. CT may be more accurate in Marchant and lateral views. However, radiography was used in this study to compare these parameters for patellar instability preoperatively and at the final follow-up examination postoperatively.

## Conclusion

Similar to MPFLR for recurrent case, MPFLR with AMZ of the TTO can improve the clinical and radiographic findings in severe cases, such as an excessive TT-TG of recurrent dislocation, preoperative OA change in the PF joint, and habitual dislocation. The remaining parameters of patellar instability could be affected for OA changes after MPFLR, with or without AMZ of the TTO, for patellar instability.

## Data Availability

Data associated with this study is retained at the Department of Orthopaedic Surgery, Osaka Metropolitan University Graduate School of Medicine. If there are any questions, please contact the corresponding author.

## Abbreviations

MPFLR: Medial patellafemoral ligament reconstruction
iMPFLR: isolated MPFLR
AMZ: anteromedialization
TTO: tibial tubercle osteotomy
PFOA: patellofemoral osteoarthritis
TT-TG: tibial tubercle-trochlear groove
ISR: Insall–Salvati ratio
